# LiteNet: Lightweight Neural Network for Detecting Arrhythmias at Resource-Constrained Mobile Devices

**DOI:** 10.3390/s18041229

**Published:** 2018-04-17

**Authors:** Ziyang He, Xiaoqing Zhang, Yangjie Cao, Zhi Liu, Bo Zhang, Xiaoyan Wang

**Affiliations:** 1 Collaborative Innovation Center for Internet Healthcare, Zhengzhou University, 75 University North Road, Erqi District, Zhengzhou 450000, China; zyhe@ha.edu.cn (Z.H.); xqzhang@ha.edu.cn (X.Z.); 2 School of Software Engineering, Zhengzhou University, 97 Culture Road, Jinshui District, Zhengzhou 450000, China; zhangbo2050@zzu.edu.cn; 3 Department of Mathematical and Systems Engineering, Shizuoka University, 5-627, 3-5-1 Johoku Hamamatsu 432-8561, Japan; liu@ieee.org; 4 College of Engineering, Ibaraki University, 4-12-1 Nakanarusawa, Hitachi, Ibaraki 316-8511, Japan; xiaoyan.wang.shawn@vc.ibaraki.ac.jp

**Keywords:** deep learning algorithms, lightweight neural network, resource-constrained mobile devices, electrocardiogram

## Abstract

By running applications and services closer to the user, edge processing provides many advantages, such as short response time and reduced network traffic. Deep-learning based algorithms provide significantly better performances than traditional algorithms in many fields but demand more resources, such as higher computational power and more memory. Hence, designing deep learning algorithms that are more suitable for resource-constrained mobile devices is vital. In this paper, we build a lightweight neural network, termed LiteNet which uses a deep learning algorithm design to diagnose arrhythmias, as an example to show how we design deep learning schemes for resource-constrained mobile devices. Compare to other deep learning models with an equivalent accuracy, LiteNet has several advantages. It requires less memory, incurs lower computational cost, and is more feasible for deployment on resource-constrained mobile devices. It can be trained faster than other neural network algorithms and requires less communication across different processing units during distributed training. It uses filters of heterogeneous size in a convolutional layer, which contributes to the generation of various feature maps. The algorithm was tested using the MIT-BIH electrocardiogram (ECG) arrhythmia database; the results showed that LiteNet outperforms comparable schemes in diagnosing arrhythmias, and in its feasibility for use at the mobile devices.

## 1. Introduction

Cardiovascular diseases (CVDs) are the main cause of mortality in the world. The World Health Organization (WHO) reported that the total number of people who died from CVDs was approximately 17.5 million in 2012, and 17.7 million in 2015, and that the total number of deaths due to CVDs continues to grow every year [1]. Therefore, CVDs pose a great threat to human health. CVDs mainly consist of arrhythmias, high blood pressure, coronary artery disease, and cardiomyopathy [2]. The electrocardiogram (ECG) is a standard piece of equipment for testing for arrhythmias; however, handling a large number of ECG samples manually is laborious and time-consuming. A computer-aided arrhythmia diagnosis system [3] on carry-on mobile devices can automatically notify patients in real-time, thus improving the efficiency of daily arrhythmia detection.

In recent years, smart mobile devices, such as smart watches, mobile phones and other wearable devices, have become more and more popular, resulting in the development of a large number of sensors and mobile applications. Nowadays, various mobile devices start to equip ECG sensors for ECG recording. Most mobile devices nowadays are still limited in both computational power and memory capacity, and are thus unfit for existing resource-demanding data analysis approaches. Therefore, in existing approaches, mobile devices often simply collect and store ECG data for analysis by human experts at a later point in time. Automatic analysis schemes try to transmit these signals to a remote server for analysis, which may easily overwhelm the limited network bandwidth of mobile devices and cause a prolonged delay. Therefore, designing an automatic analysis algorithm that runs on resource constrained mobile devices or on the network edge [4,5,6,7,8,9,10] can help reduce both human labor and response time, with virtually no network bandwidth consumption; hence, the development of this technology is of fundamental importance. In this paper, we address real-time automatic arrhythmia detection by investigating an ECG arrhythmia detection mechanism that runs on the resource-constrained mobile devices. We propose a light-weight edge computing algorithm based on deep-learning for such task.

Deep-learning based classification algorithms [11], which often outperform traditional algorithms substantially, are emerging and are more and more widely supported by the information technology society [12]. They have been successfully applied in many fields, such as image classification, speech and natural language processing, scene labeling, etc. Deep-learning based algorithms are useful for identifying different wave types and the complicated relationships among them in time series. Furthermore, deep-learning based algorithms outperform hand-made feature extraction methods assembled with traditional classifiers, and can achieve equivalent accuracies for both noise-free and noisy data. Therefore, numerous deep learning models have been proposed as useful tools for arrhythmia detection in ECG signals [13,14]. These models range from simple feed-forward networks and back-propagation neural networks to complex networks such as convolutional neural networks (CNNs) and recurrent neural networks (RNNs). The computational complexity of deep-learning- based algorithms in both training and working phases is highly strongly related to algorithm scale. To our knowledge, most deep-learning- based algorithms mainly focus on improving arrhythmia detection accuracy, while paying little attention to algorithm scale or model size reduction (e.g., memory and computational power requirements), leading to these models being poorly suited not suitable forfor resource-constrained mobile devices. Therefore, designing a light-weight, deep-learning based algorithm for resource-constrained mobile devices with comparable accuracy remains a challenge. Various strategies are have recently been used to build small and efficient neural networks. Inspired by GoogleNet [15], SqueezeNet [16] and MobileNets [17], we devised a light-weight, CNN-based neural network for resource-constrained mobile devices, namely LiteNet, for accurately diagnosing arrhythmias in real-time. LiteNet focuses on balancing the tradeoff between the model size and the accuracy of the output. In summary, the contributions of this paper are as follows:

- We propose a light-weight neural network model, named LiteNet, which can not only be trained faster than traditional deep learning algorithms on remote servers, but which is also dramatically more resource-friendly when working on mobile devices. LiteNet can therefore be trained on low-capacity servers and the trained model can be installed on resource-constrained mobile devices for arrhythmia detection with low resource consumption.
- Filters with heterogeneous sizes in each convolutional layer are designed to get various feature combinations, in order to achieve high accuracy. Both the sizes of each filter and the total number of filters can be adapted within each convolutional layer, which helps substantially in obtaining different feature maps in a convolutional layer.
- LiteNet verifies that Adam optimizer [18] can be used as a stochastic objective function. It can improve the accuracy of the model compared with the traditional gradient descent optimizer while requiring minimal parameter tuning in the training process.
- We conducted extensive experiments to evaluate the performance of LiteNet in terms of both accuracy and efficiency. Experimental results confirm that LiteNet outperforms recent state-of-the-art networks in that it achieves comparable or even higher accuracy with much higher resource-efficiency. LiteNet is thus well suitable for resource-constrained mobile devices.

The remaining of this paper is organized as follows. Section 2 discusses related work. Section 3 introduces the algorithm of LiteNet. The experiments and results are elucidated in Section 4. Finally, we present our conclusions in Section 5.

## 2. Related Work

We next briefly reviewed related work in edge computing and arrhythmia detection. For each subject we highlight the differences between existing approaches and LiteNet.

### 2.1. Edge Processing

Recently, the number of mobile applications that are running on mobile devices has increased drastically; this has promoted the development of mobile edge computing (MEC), including the connected base station and mobile devices. By running applications and services closer to the user, MEC brings many advantages, such as shorter response time and reduced network traffic. However, compared to the traditional cloud, computational resources such as the CPU cycle and memory, are limited. To address this situation, some research [19,20] has focused on how to perform optimal resource allocations on resource-constrained mobile devices, and conducted MEC in various scenarios [21,22,23].

**Figure 1:**
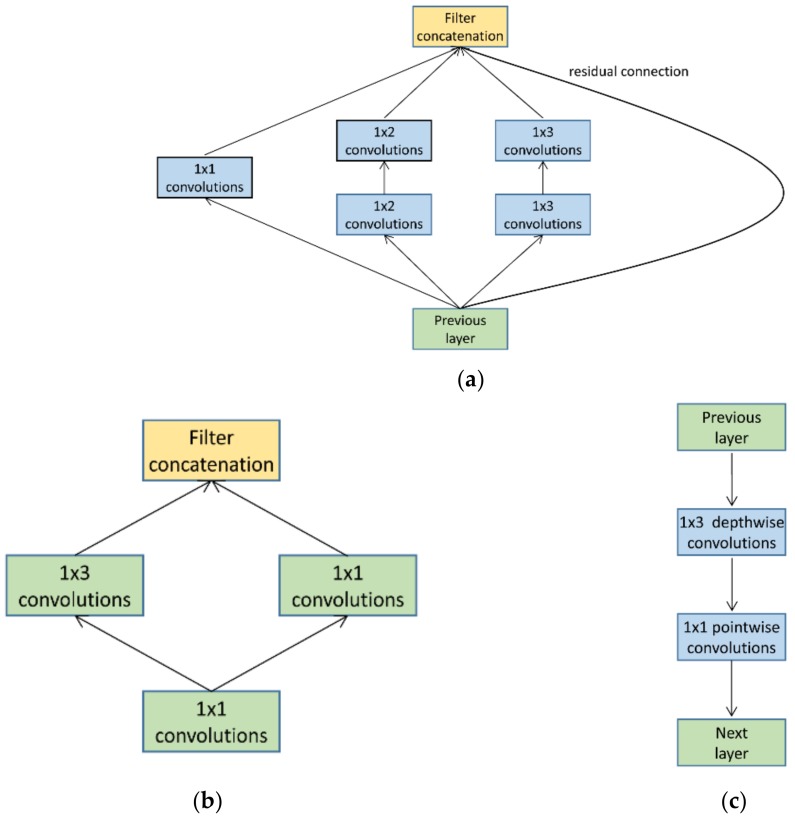
(**a**) Inception module; (**b**) Fire module; (**c**) Depthwise separable convolution that factorizes a standard convolution into a depthwise convolution and a pointwise convolution.

The edge processing algorithms play a crucial role in providing efficient MEC. Although deep-learning based algorithms usually have excellent performance, they demand high computational capability and large memory. Hence, the traditional deep learning algorithms are too heavy for the mobile devices. In the literature, there has been an emerging interest in designing light-weight deep learning models. GoogleNet introduced the Inception module, shown in Figure 1a, for better feature extraction at low computational cost. Convolution kernels of heterogeneous and small sizes (1 × 2, 1 × 3 and 1 × 1) are combined in this module. However, if directly applied to the mobile devices, this module may overwhelm the memory capacity of the mobile devices due to its large feature maps. In contrast, the Fire module in SqueezeNet, as shown in Figure 1b, focuses on reducing the parameter volume by limiting the number of feature maps. SqueezeNet replaces a standard convolution layer with two specially designed layers: squeeze layer and expand layer. Squeeze layers use filters of size 1 × 1 and the number of filters are at least 2× less than the preceding layer, while expand layers use a mixture of filters of size 1 × 1 and 1 × 3 for certain accuracy guarantee in a parameter-volume-controlled manner. We adapt the 1 × 1 squeeze layer in LiteNet for parameter compression, and term it as *squeeze convolution layer* in our work. In MobileNets, Andrew et al. used a depthwise separable convolution [24] strategy that factorizes a standard convolution into a depthwise convolution and a pointwise convolution as shown in Figure 1c. Depthwise convolution applies each convolution kernel to each feature map, while pointwise convolution is used to combine the output of the preceding layer. Such structure can therefore effectively reduce at least 2× computational load comparing to standard convolutions. 

**Figure 2:**
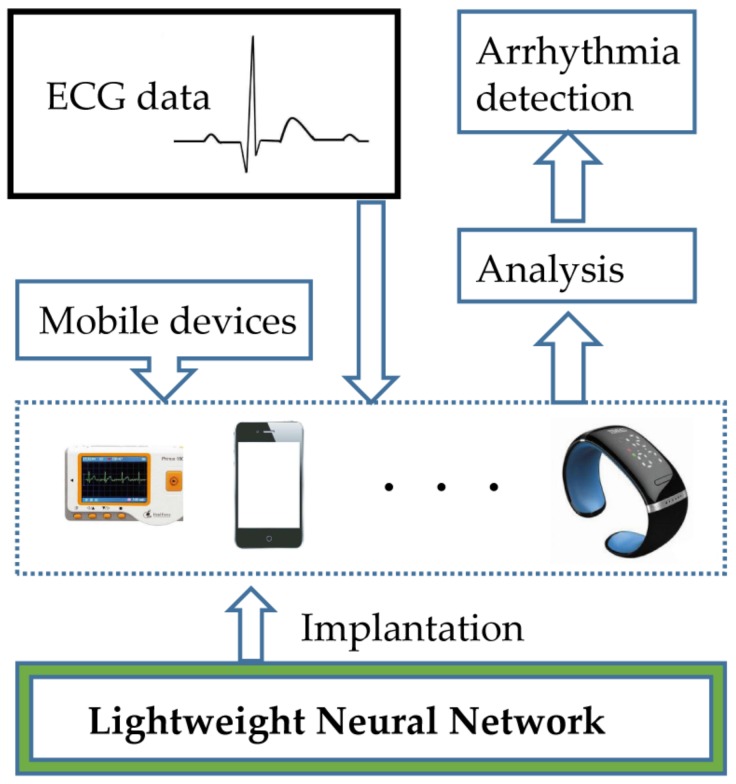
System illustration of arrhythmia detection based on ECG using LiteNet.

In this paper, the LiteNet combines the strengths of these modules, to achieve high feature extraction ability while maintaining a low computational cost by parameter volume compression. We describe LiteNet in detail, which applies lightweight deep-learning based algorithms to identify arrhythmias. Figure 2 shows, an example of the deep learning scheme for resource-constrained mobile devices. As shown in Figure 2, firstly, resource-constrained edge devices collect ECG data from the users through smart sensors, then the LiteNet model is deployed on resource-constrained mobile devices (mobile phone, Smartwatch and ECG monitor) and used to detect different arrhythmias with collected data, finally, the LiteNet model analyzes ECG data and produces arrhythmia identification result to the users in real time.

### 2.2. Arrhythmia Detection

Arrhythmia detection using ECG is an important research topic and numerous algorithms in both traditional machine learning and deep learning aspects have been developed.

With conventional machine learning, the diagnosis of arrhythmias using ECG requires several processes: data preprocessing, feature extraction, normalization and classification. Due to the existence of baseline drift and ECG noise (e.g., muscle motion), it is vital for traditional machine learning methods to perform efficient and accurate de-noising operations before feature extraction can be employed. Common solutions, such as the low-pass linear-phase filter, high-pass linear-phase filter, median filter, and mean median filter, are usually used for such de-noising task. Classical feature extraction approaches, such as continuous wavelet transform (CWT) [25], S-Transform (ST), discrete Fourier transform (DFT), principal component analysis (PCA), Daubechies wavelet (Db4) [26], and independent component analysis (ICA) [27] can then be applied. Researchers in [28] used three machine-learning based algorithms, namely, Discrete Wavelet Transform (DWT) [29], Empirical Mode Decomposition (EMD) [29] and Discrete Cosine Transform (DCT) to obtain coefficients from ECG signals. Then, the researchers adopted the Locality Preserving Projection (LPP) method to reduce the number of these coefficients and used the F-value measure to rate the LPP features. Finally, the best coefficients were fed into the K-Nearest Neighbor (KNN) model for arrhythmias diagnosis. The results from the experiments proved that the machine-learning-related algorithms that they devised achieved excellent performance. Similarly, Personalized Features Selection and Support Vector Machines were used to identify arrhythmias in [30,31], respectively. They are able to extract features accurately and achieve good results. However, traditional machine learning approaches to classify ECG data usually requires complex data preprocessing, thus embedding them into mobile devices increase heavy workload on the device.

In deep learning aspect, Pranav Rajpurkar et al. [13] devised a 34-layer convolutional neural network (CNN) for arrhythmia detection with a single-lead ECG signals. They compared the model performance with the performances of cardiologists, and showed that the proposed model outperformed the cardiologists. One explanation for their excellent results is that they adopted the residual connection strategy to alleviate degradation problem. Yi Zheng et al. [32] introduced a multi-channel convolutional neural network (MCNN) for detecting arrhythmias using multi-lead time-series ECG signals. They adopted two-lead ECG signals to test the MCNN model and the results from the experiments showed that an accuracy of 94.67% was achieved. Overall, the deep learning schemes proposed for ECG diagnosis exhibit excellent performance. Rajendra Acharya et al. [33] compared the performance of CNN for noisy and noise-free ECG datasets using a publicly available arrhythmia database. The results from the experiments show that the model achieved the same arrhythmia detection accuracy for noisy and noise-free ECG signals. This proves that the removal of noise is not necessary in deep learning algorithms for ECG diagnosis. Most of the current deep learning models focus on improving accuracy and often resulting in the models too large to be embedded in mobile devices. 

## 3. Method of LiteNet

In this section, we first introduce the One-Dimensional Convolution Kernel designed for LiteNet. We then describe core modules of LiteNet, as well as the overall architecture, followed by the introduction of the Adam optimizer, adapted for the back propagation training process.

### 3.1. One-Dimensional Convolution Kernel

CNN is a well-known deep learning architecture inspired by the natural visual perception mechanism of living creatures. Classic CNN consists of cascaded convolutional layers and pooling layers. Each convolutional layer calculates the inner product of the linear filter and the underlying receptive field of an input segment, and applies a nonlinear activation function. The resulting outputs are called feature maps. The pooling layer is a vital component of CNN. It reduces the computational cost by cutting connections between convolutional layers.

However, CNN is designed for two-dimensional input such as image pixels. For a one-dimensional ECG time-series signals, its convolution kernel needs to be adapted. We introduce the modified equation for one-dimensional convolution as:

(1) $$\begin{array}{cc} y[n] & =x[n]*h[n],\\ & =\sum_{k=0}^{m-1}x[k]h[n-k], \end{array}$$

where *x*[*n*] is the input sequence of length m, *h*[*n*] is the kernel sequence and *y*[*n*] is the output sequence. Our proposed LiteNet adopts Equation (1) as the kernel function. For example, the length of *x*[*n*] is 3, the length of *h*[*n*] is 3, so the length of output is 5. The input sequence is *x*[*n*] = [*x*_1_, *x*_2_, *x*_3_], the kernel sequence is *h*[*n*] = [*h*_1_, *h*_2_, *h*_3_]. *h* (−*k*) is to reverse the sequence of *h*(*k*), while *h*(*n*–*k*) is to translate the *h*(−*k*) to n points. The output sequence therefore is,

(2) $$\begin{array}{l} y[0]=x[0]h[0-0]+x[1]h[0-1]+x[2]h[0-2]=h_{1}*x_{1}\\ y[1]=x[0]h[1-0]+x[1]h[1-1]+x[2]h[1-2]=h_{1}*x_{2}+h_{2}*x_{1}\\ y[2]=x[0]h[2-0]+x[1]h[2-1]+x[2]h[2-2]=h_{1}*x_{3}+h_{2}*x_{2}+h_{3}*x_{1}\\ y[3]=x[0]h[3-0]+x[1]h[3-1]+x[2]h[3-2]=h_{2}*x_{3}+h_{3}*x_{2}\\ y[4]=x[0]h[4-0]+x[1]h[4-1]+x[2]h[4-2]=h_{3}*x_{3} \end{array}$$

**Figure 3:**
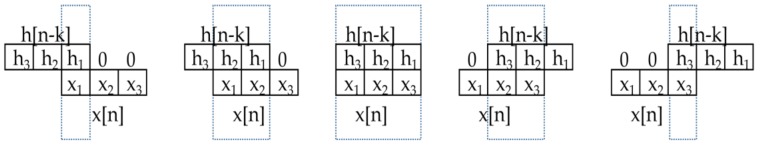
One-dimensional convolution process.

The concrete convolution process is explained in Figure 3.

### 3.2. Lite Module

**Figure 4:**
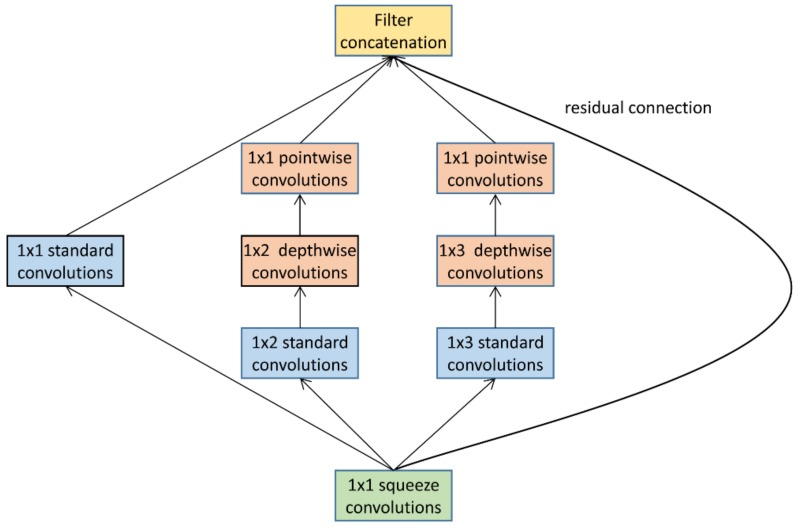
Lite module.

In this paper, we devise an efficient CNN microarchitecture, which is named the Lite module, as shown in Figure 4. It constitutes the core layers of LiteNet. The Lite module consists of a 1 × 1 squeeze convolutional layer and a variant of the inception module.At the bottom, the squeeze convolutional layer (green) has filter of size 1 × 1. It is a variant of the inception module and the current design of the modified inception module is restricted to filter sizes of 1 × 1, 1 × 2 and 1 × 3. The key motivation for using a small filter size is reduced computational cost between convolutional layers. Furthermore, it uses two different convolution strategies: standard convolutions (blue), and depthwise/pointwise convolutions. Additionally, an optional residual connection [34] is adopted in the Lite module. The lite module has the following advantages:

- It can reduce the parameter volume efficiently. It relies heavily on a squeeze convolutional layer and a depthwise separable convolutional layer, which cuts down on the parameter volume.
- The single 1 × 1 standard convolutional layer is able to enhance abstract representations of local features and cluster correlated feature maps [35].
- Large activation maps can be generated by Lite modules due to postponed down-sampling, which contributes to the high accuracy of the results.
- The Lite module contains filters of heterogeneous size, which facilitates the exploration of different feature maps for key feature extraction.
- The optional residual connection can eliminate the effect of the gradient vanishing problem in deep networks.

### 3.3. LiteNet Architecture

**Figure 5:**
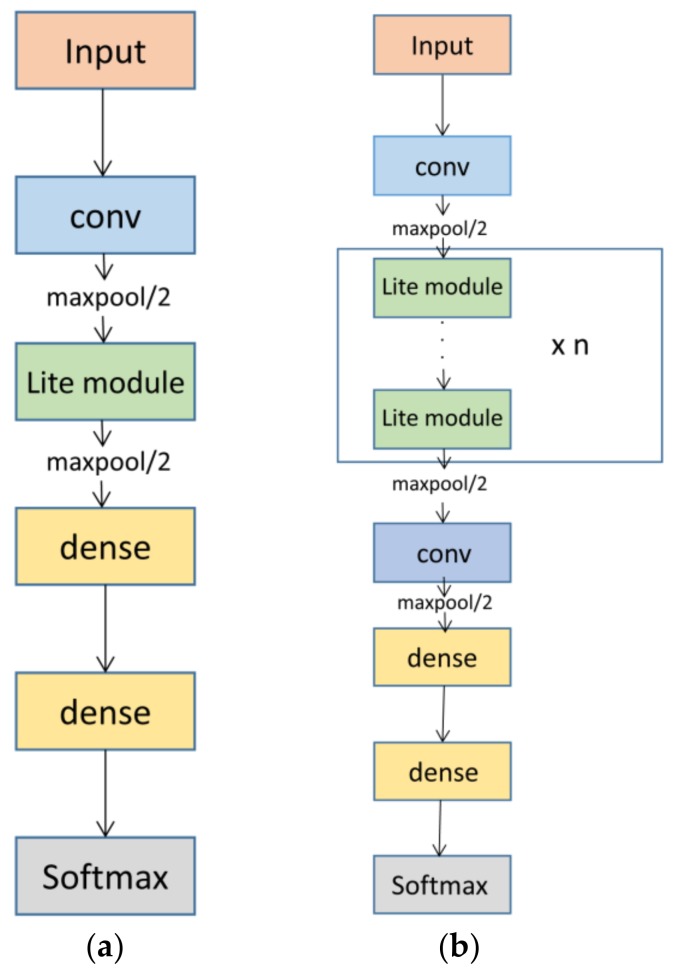
LiteNet Architecture: (**a**) basic LiteNet architecture; (**b**) extended LiteNet architecture.

Now that the Lite module has been introduced, we describe the LiteNet architecture in detail. LiteNet is built from Lite module layers, which represent an efficient convolution module design approach, as described above. They can reduce sharply both the parameter volume and the computational cost. As illustrated in Figure 5, a basic LiteNet model consists of a single Lite module, which we use in this study, as shown in Figure 5a. An extended LiteNet model contains a stack of Lite modules, as shown in Figure 5b.

LiteNet takes as input a time series of ECG signals. The basic network begins with a standard convolutional layer, followed by one Lite module, two fully connected layers (dense) and a softmax layer. For multi-class problems in deep learning, it is standard to use softmax as a classifier [36]. This study, arrhythmias detection has 5 possible classes, the softmax layer has 5 units represented by *p_i_*, where *i* = 1, …, 5. *p_i_* denotes a probability distribution. Therefore

(3) $$\sum_{i}^{5}p_{i}=1$$

*x* is the output of the upper-layer unit, *W* is the weight connecting upper-layer to the softmax layer, the total input into softmax layer, given by *z*, is

(4) $$z_{i}=\sum_{k}x_{k}W_{ki}$$

The softmax layer calculates the final likelihood of each output and is computed as follows:

(5) $$p_{i}=\frac{\exp(z_{i})}{\sum_{j}^{5}\exp(z_{j})}$$

The predicted class $\hat{i}$ would be

(6) $$\begin{array}{cc} \hat{i} & =\arg\max p_{i}\\ & =\arg\max z_{i} \end{array}$$

Furthermore, we use cross-entropy function (loss function) to determine how close the actual output is to the expected output. The smaller the value of cross-entropy, the closer the two probability distributions are:

(7) $$H(p,q)=-\sum_{x}p(x)\log q(x)$$

*H*(*p*, *q*) is cross-entropy, *p* and *q* represent expected output and actual output, respectively. *p* (*x*) and *q*(*x*) represent their probability distribution. For example, *N* = 3, expected output *p* = (*p*_1_, *p*_2_, *p*_3_), actual output *m* = (*m*_1_, *m*_2_, *m*_3_), *n* = (*n*_1_, *n*_2_, *n*_3_), Therefore

(8) $$\begin{array}{l} H(p,m)=-(p_{1}*\log^{m_{1}}+p_{2}*\log^{m_{2}}+p_{3}*\log^{m_{3}})=k_{1}\\ H(p,n)=-(p_{1}*\log^{n_{1}}+p_{2}*\log^{n_{2}}+p_{3}*\log^{n_{3}})=k_{2} \end{array}$$

if *k*_1_ is less than *k*_2_, *k*_1_ closer to expected output, otherwise *k*_2_ closer to expected output. But at real run time, these are all M*N matrices. *M* represents the number of batch and *N* represents the number of classification. All in all, the smaller cross-entropy, the better classification result performance gets.

For a specified accuracy, we apply several activation functions, e.g., tanh, sigmoid, rectifier linear unit, and leaky rectifier linear unit (LeakyRelu) [37], to basic LiteNet and find that the LeakyRelu activation function outperforms the other activation functions. Therefore, LeakyRelu is used as the activation function for both convolutional layers and dense layers. The number of filters per Lite module can be adapted, depending on either the size of the input or the accuracy.

Table 1 summaries the structure of basic LiteNet. The key characteristics of different layers in basic LiteNet are detailed as follows:

- Standard convolutional layer: Most CNN-based architectures begin with few feature maps and large filter size. We also use this design strategy in this paper. A standard convolutional layer has five filters and convolves with a filter size of 1 × 5.
- Max-pooling layer: LiteNet performs two max-pooling operations with a stride of two after a standard convolutional layer and Lite module layer. The max-pooling operation can lower the computational cost between convolutional layers.
- Lite module layer: The use of a small filter size can reduce the computational cost and enhance the abstract representations of local features in a heterogeneous convolutional layer. The Lite module has filter sizes of 1 × 1, 1 × 2 and 1 × 3 for this purpose, as shown in Figure 4, and the feature map settings of the Lite module layer are listed in Table 1.
- Fully connected layers: Two fully connected layers are used in basic LiteNet and in most deep learning architectures. The first and second layers consist of 30 and 20 units, respectively, which can yield expected classification result performance.
- Dropout [38] layer: To tackle the overfitting problem, the dropout technique is adopted. We build a dropout layer after the two dense layers and set the dropout rate at 0.3.
- Softmax layer: The softmax layer has five units. The softmax function is used as a classifier to predict five classes.

### 3.4. Adam Optimizer

The training process of LiteNet is carried out by a backpropagation [39] approach. Stochastic gradient descent optimization is of vital practical importance in the backpropagation training process for deep learning models. Conventional stochastic gradient descent algorithms usually require special tuning tricks and large memory during the training process. It is time-consuming, laborious and difficult to set optimal hyper-parameters for deep learning models. To initialize hyper-parameters easily with little tuning during the training process, we adopt the Adam optimizer [22], which is a first-order gradient-based descent optimizer of stochastic objective functions that is based on adaptive estimates of lower-order moments and computes individual adaptive learning rates for different hyper-parameters from estimates of first and second moments of the gradients. It is very easy to implement and computationally efficient. This approach can also improve the classification performance compared with classic gradient descent algorithms and requires little memory and little tuning in the training process. 

## 4. Experiments and Results

### 4.1. Dataset and Data Processing

According to the Association for the Advancement of Medical Instrumentation (AAMI) [40], non-life-threatening arrhythmias can be divided into 5 main classes: N (normal beat), S (supraventricular ectopic beat), V (ventricular ectopic beat), F (fusion beat), and Q (unknown). In this paper, we used the datasets from the MIT-BIH Arrhythmia database [41]. This database consists of 48 half-h-long ECG recordings of Lead II ECG signals at a sample rate of 360 Hz from 48 subjects. Furthermore, these recordings were annotated by at least two cardiologists. 109,449 ECG samples are extracted from this database. The numbers of five classes samples are 90,952, 2781, 8039, 7235 and 802, respectively. Two datasets are extracted from the MIT-BIH Arrhythmia database: dataset A (set A) and dataset B (set B). Since a single heartbeat is normally between 1 and 2 s, we split the ECG data into 1-s periods to generate set A, and generate set B with 2-s-period split. We test the performance of LiteNet by both sets. To perform such split, we first recognize each R-peak, and take uniform-length signal sections both proceeding and following R-peak. 180 samples and 360 samples period on both sides of an R-peak are selected for a data piece of set A and set B respectively. Therefore, the length of each ECG sample of set A and set B are 360 and 720 respectively. Both datasets consist of original data (including noise) to simulate the real arrhythmia detection.

LiteNet is utilized to automatically and efficiently detect the five classes of arrhythmias on the wearable medical equipment with less overhead. Both set A and set B will be used for training and assessing LiteNet. However, the five classes are imbalanced in both set A and set B. To overcome the imbalance of two datasets in the five classes, a data synthesis strategy is used. We use the starting point of translation and plus-noise strategies to synthetize data. After augmentation, the number of samples in each class is 93,000 and the total number of ECG segments that include five classes has been increased to 465,000. Each ECG segment is normalized by using the Z-score method to solve the problem of amplitude scaling and eliminate the offset effect.

**Figure 6:**
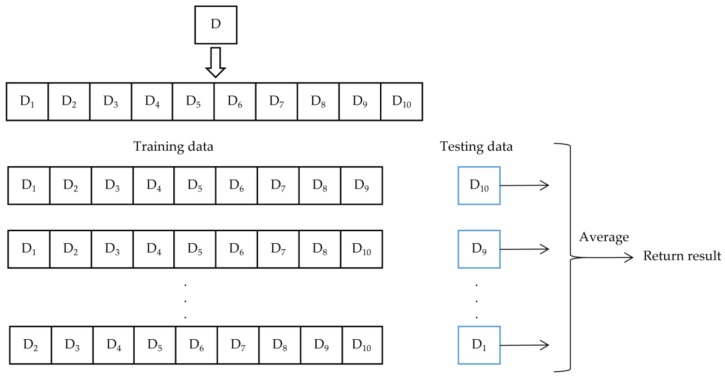
Ten-fold cross-validation.

Furthermore, we used the ten-fold cross-validation method which is a common measure in both traditional machine learning and deep learning to get some evaluations on data. The ECG datasets were divided into 10 independent folds. The number of samples in each fold is equal. Each time, 9 folds are used for training and the remaining fold is used as the testing dataset, with no data intersection. This is repeated ten times at the same learning rate and return average evaluations of ten results as shown in Figure 6. Each time, the training dataset and testing dataset are different in ten-fold cross-validation, and can be summarized as follows:

(9) $$\begin{array}{l} D=D_{1}⋃D_{2}⋃\ldots ⋃D_{10},\\ D_{i}⋂D_{j}=\emptyset (i\neq j) \end{array}$$

*D* represents the total ECG data, *D_i_* and *D_j_* represent independent subset. 

### 4.2. Experimental Setup

We used the Scikit-learn [42] library and TensorFlow [43] to implement LiteNet. We manually set the learning rate at 0.005, initialized the weights from scratch and used default settings for other hyper-parameters since the Adam optimizer was used in the training process. We experimented with batch sizes that ranged from 20 to 150 and finally set the batch size at 50. To evaluate the performance of the Adam optimizer, we also train LiteNet with the stochastic gradient descent (SGD) optimizer, which is a classic and efficient optimization method for the backpropagation training process. The training and testing of LiteNet were repeated 20 times to avoid occasion. 

To test the performance of LiteNet, we use two sets (set A and set B) to train the same LiteNet architectures. The five models are trained on a server with six Intel Xeon (R) at 2.60 GHz (E5-2650) processors and 64 GB memory and test the running time of five models on a normal personal computer with Intel (R) CPU i3-2370M at 2.40 GHz and 4 GB memory (which is similar with many MEC computing environment) without any engineering optimization. To make the experiments realistic, noise is not removed from the ECG signals in advance. 

### 4.3. Baselines and Metrics

For comparison, four state-of-the-art CNN are used as baselines, namely, AlexNet [44], GoogleNet, SqueezeNet and MobileNets. AlexNet is built with standard convolutions, GoogleNet is built with Inception modules, SqueezeNet is built with Fire modules and MobileNets is built with depthwise separable convolution. AlexNet has 3 standard convolutional layers. The layers are convolved with kernels of sizes (1 × 5, 1 × 3, 1 × 3) and have 5, 10 and 20 filters, respectively. GoogleNet, SqueezeNet and MobileNets all begin with a single standard convolutional layer with 10 filters (kernel size of 1 × 5). We apply the inception module, fire module and depthwise separable convolution strategies, as shown in Figure 2, to GoogleNet, SqueezeNet and MobileNets, respectively. Each of the convolutional layers in the inception module has 8 filters. One inception module is used and an additional 1 × 2 standard convolutional layer with 10 filters is used in GoogleNet. Two identical fire modules are used in SqueezeNet. The squeeze layer has 3 filters and the expand layer has 8 filters in the fire module. MobileNets uses two depthwise separable convolutional layers with 10 and 15 filters. The four comparison models also use Adam as a stochastic objective function.

Table 2 shows the schematic diagram of confusion matrix. We use the following metrics to evaluate our model performance:

- Parameter Count (PC): This metric can be loosely defined as the total parameter volume, except the number of fully connected layers. In deep learning, PC represents the model size and the number of unit connections (computational cost) between layers. PC is an important factor of computational complexity of deep-learning based algorithms. The lower PC is, the lower the computational cost and the less memory the model needs.
- Accuracy (ACC): ACC is an overall metric that measures the correctness of classification into the arrhythmias classes relative to all examples [45].
  (10) $$\mathrm{ACC}=\frac{\mathrm{TP}+\mathrm{TN}}{\mathrm{TP}+\mathrm{FN}+\mathrm{FP}+\mathrm{TN}}$$
- F-measure (F1): F1-measure is a measure that combines precision and recall and is equal to the harmonic mean of precision and recall. Higher F1 indicates more effective classification performance.
  (11) $$\begin{array}{l} \mathrm{Precision}=\frac{\mathrm{TP}}{\mathrm{TP}+\mathrm{FP}} \end{array}$$
  (12) $$\begin{array}{l} \mathrm{Recall}=\frac{\mathrm{TP}}{\mathrm{TP}+\mathrm{FN}} \end{array}$$
  (13) $$F1=\frac{2*\mathrm{precision}*\mathrm{recall}}{\mathrm{precision}+\mathrm{recall}}$$
- Area under the Curve (AUC): The AUC value is equivalent to the probability that a randomly chosen positive example is ranked higher than a randomly chosen negative example. The corresponding AUC values of classification performance evaluations are shown in Table 3.

### 4.4. Experimental Results

Table 4 and Table 5 present the classification performances of Adam optimization and stochastic gradient descent (SGD) on set A and set B, respectively. LiteNet with Adam increases the accuracy by at least 1% compared to LiteNet with SGD. Since Adam provides better results than SGD, we use Adam optimizer in the following experiments.

**Figure 7:**
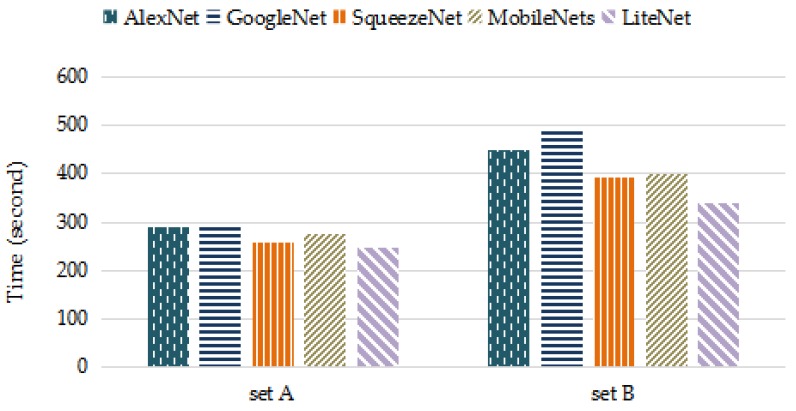
Training time of five models.

**Figure 8:**
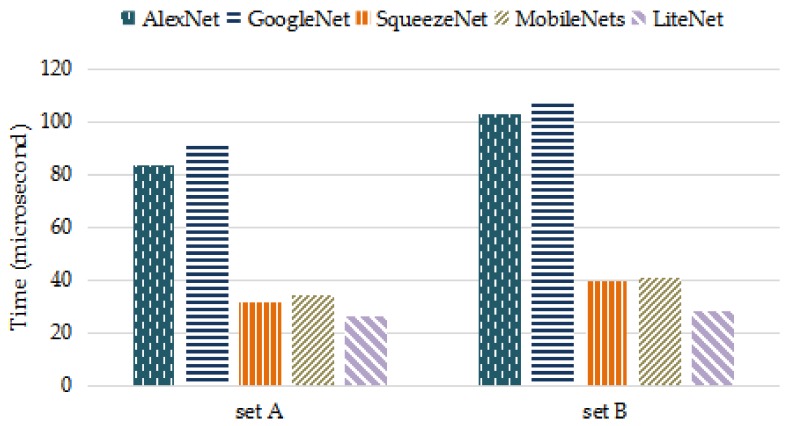
Testing time of five models.

Table 6 and Table 7 present the performances of the LiteNet model and the four comparison models on set A and set B, respectively. For PC, LiteNet decreased about 10% PC than MobileNets and SqueezeNet, while AlexNet and GoogleNet use twice as many PC than LiteNet. The computational complexity of deep-learning based methods in both training and working phases is closely related to PC. Smaller PC can make the algorithms deployed on resource-constrained mobile devices to carry out timely task easily. Furthermore, SqueezeNet and MobileNet have been successfully deployed on resource-constrained mobile devices, such as mobile phones, robotics and self-driving car. LiteNet has smaller parameter volume compared to MobileNet and SqueezeNet, which ensures a lower computational cost and also can be deployed on resource-constrained mobile devices. For accuracy, the basic LiteNet model achieved the expected accuracies of 97.87% and 98.80% on sets A and B, respectively. From the result we conclude that longer data period produces higher accuracy, since the data within each period in set B contains a complete heartbeat signal, instead of partial data per heartbeat present in set A. LiteNet slightly outperforms SqueezeNet and MobileNet. The AUC measure is also used to assess the model performances. LiteNet achieved AUC values of 97.78% and 99.30%. According to Table 3, LiteNet is considered as an excellent classifier in random conditions. There are several explanations for the performance of LiteNet: the Adam optimizer is used in the training process, and the Lite module uses the postponed down-sampling strategy. Figure 7 presents the training times of the five models in the training phase. LiteNet achieved the best performance, as we expected. LiteNet required less training time than the other four models. Figure 8 presents the average testing time of the five models on all testing samples with cross-validation method and LiteNet uses less testing time than compared models. Although LiteNet, SqueezeNet and MobileNet use similar time on set A, it is obvious that LiteNet uses shorter time to produce arrhythmia detection results than SqueezeNet and MobileNet on set B. According to Figure 8, it can be inferred that the longer data is, the less time LiteNet uses to produce results than compared models more visibly. There are several reasons for testing time results. Firstly, the data is longer, more sliding convolution operations are required in a convolution layers (the longer data is, the more computing time requires). LiteNet uses 1 × 1 convolution squeeze layers and depthwise and pointwise convolution layers, which both can reduce computational complexity (testing time) sharply as introduced in Section 2.1, while SqueezeNet only uses 1 × 1 convolution squeeze layers and MobileNet just uses depthwise and pointwise convolution layers. Secondly, LiteNet uses one less pooling layers than both SqueezeNet and MobileNet, which reduce a step to process data.

**Figure 9:**
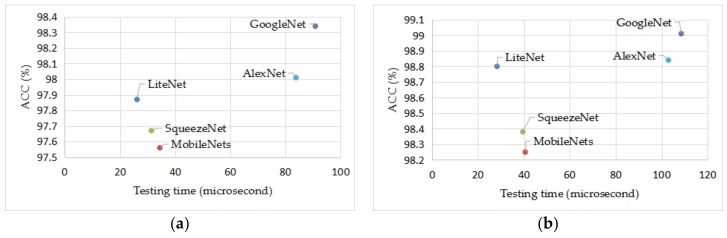
The trade-off between accuracy and testing time on five models. (**a**,**b**) represent dataset A and dataset B, respectively.

According to Table 6 and Table 7 and Figure 8, LiteNet uses about twice PC less than GoogleNet and AlexNet, the testing time of LiteNet is about four-fold faster than both GoogleNet and AlexNet. When comparing to both SqueezeNet and MobileNet, PC of LiteNet decreased by approximately 10%, the testing time of LiteNet decreased by approximately 30%. Accuracy of LiteNet improves 0.35% and 0.42% than SqueezeNet and MobileNet on set A and improves 0.51% and 0.60% than SqueezeNet and MobileNet on set B. In order to evaluate accuracy and testing time visibly. Figure 9 shows the trade-off between ACC and testing time on the five models. LiteNet is unable to highlight the obvious advantages due to the short sample size of data set A. When testing with dataset B, the set of 2 s heartbeat samples, LiteNet achieves comparable accuracy with much lower testing time comparing with conventional CNN networks such as GoogleNet and ALexNet. For dataset A, due to the amplified impact of partial hearbeat samples to the much smaller parameter and feature volume, LiteNet performs not as good as that of dataset B and that of conventional CNN networks in terms of accuracy. Nevetheless it outperforms conventional CNN networks in testing time by a large margin, while surpasses MobileNet and SqueezeNet in terms of accuracy. Overall, LiteNet is capable of using the model size efficiently and receiving excellent classification result performance and can be deployed mobile devices.

**Figure 10:**
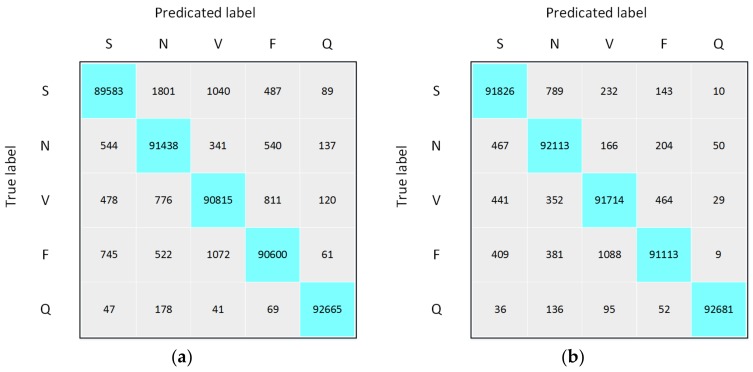
Confusion matrixs for LiteNet on set A (**a**) and set B (**b**).

To better understand the misclassified cases and overall classification performance of LiteNet, we present confusion matrix graphs of LiteNet on set A and set B in Figure 10. The number of true labels for each class is equal after data preprocessing. This number can be more convincing to validate whether the model is good or bad. According to Figure 10a,b, normal beat corresponds to the worst precision results of 96.54% and 98.23%, respectively; supraventricular ectopic beat corresponds to the worst sensitivity (recall) result of 96.33% on set A, and fusion beat corresponds to the worst sensitivity (recall) result of 97.97% on set B. Based on the performance results, we summarize the benefits of LiteNet as follows:

- LiteNet is fully automatic. Hence, no additional feature extraction, selection, or classification is required.
- LiteNet has small model size. Thus, it requires less memory and has low computational cost. Small model size results in little transmission overhead when exporting new models to mobile terminals, while smaller memory footprint and low computational cost make LiteNet more feasible for mobile devices (e.g., wearable ECG monitors. As to the requirement of the wearable ECG monitor, the monitor can only have the detection (generating the ECG signal) function and sends the data to the nearby computing devices (network edge) to help calculate the generation results. Given the network edge can be personal computer, or even server, the computation capability and embedded memory is relatively larger than ECG monitors. As shown in the experimentation results, the detection can be finished in real-time with very high accuracy using normal personal computer with Intel (R) CPU i3-2370M at 2.40 GHz and 4 GB memory without any engineering optimization.).
- Although LiteNet has a small model size, a satisfactory recognition rate can be achieved.

LiteNet is not without shortcomings. Massive amounts of ECG signal data are required for training LiteNet before the model can be deployed on mobile devices. Although the training process of LiteNet is expensive, once the model training has been completed, arrhythmia detection is fast. It can be deployed on wearable medical equipment to assist cardiologists in objective diagnosis of ECG signals in real-time clinical consultations in both third-world countries and at home, where access to a cardiologist and specialized medical equipment is very sparse. It can also reduce the cost of ECG devices by decreasing the hardware configuration requirements.

## 5. Conclusions

We developed a small and efficient neural network for resource-constrained mobile devices, named LiteNet, for detecting arrhythmias automatically and efficiently, which is crucial for ECG diagnosis in Smart Health Platform. Comparing to recently proposed light-weight neural networks, i.e., MobileNets and SqueezeNet, LiteNet uses the similar parameter volume and achieves accuracies of 97.87% and 99.30% on two datasets, respectively, with original ECGs. However, resource requirements of LiteNet are much lower. Therefore, LiteNet can be deployed on a resource-constrained mobile device to monitor the heart activity and detect arrhythmias. Furthermore, LiteNet has the potential to be a useful tool that aids medical experts in diagnosing ECG signals and decreases the burden on cardiologists in polyclinics through remote diagnosis.

In the future, we will collect and annotate ECG records from real patients and adopt longer ECG signals as the input for training the model. Multi-lead ECG records will also be used to train the model to detect other forms of heart disease. On the clinical side, we will develop an ECG system that can be deployed on wearable medical equipment and low-cost ECG devices to test and verify its effects and drawbacks.

## Figures and Tables

**Table 1:** Summary of the basic LiteNet model for this work.

Layer		Kernel Size	Stride	No. of Filters
Standard Conv.		1 × 5	1	5
Max-Pooling		1 × 2	2	5
Lite Module	Squeeze Conv.	1 × 1	1	3
	Standard Conv.	1 × 1	1	6
		1 × 2	1	6
		1 × 3	1	6
	Depthwise Conv.	1 × 2	1	6
		1 × 3	1	6
	Pointwise Conv.	1 × 1	1	6
		1 × 1	1	6
Max-Pooling		1 × 2	2	18
Dense				30
Dense				20

**Table 2:** Confusion matrix principle.

		True Label	
		Normal	Arrhythmia
Predicate label	normal	True Positive (TP)	False Positive (FP)
	arrhythmia	False Negative (FN)	Ture Negative (TN)

**Table 3:** Classification evaluation and corresponding AUC values.

Evaluation	Excellent	Good	Fair	Poor	Failure
AUC range	0.9–1.0	0.8–0.9	0.7–0.8	0.6–0.7	0.5–0.6

**Table 4:** Comparison of Adam and SGD using LiteNet for set A with the ten-fold cross-validation method.

Optimizer	ACC (%)	AUC (%)	F1-Measure (%)
SGD	95.66	96.67	98.65
Adam	97.87	97.78	99.33

**Table 5:** Comparison of Adam and SGD using LiteNet for set B with the ten-fold cross-validation method.

Optimizer	ACC (%)	AUC (%)	F1-Measure (%)
SGD	96.67	97.55.	98.34
Adam	98.80	99.30	99.66

**Table 6:** Comparison of four CNN-based networks for set A with the ten-fold cross-validation method.

Network	PC	ACC (%)	AUC (%)	F1-Measure (%)
AlexNet	1100	97.89	98.48	99.35
GoogleNet	1276	98.34	98.79	99.58
SqueezeNet	528	97.53	97.28	99.09
MobileNets	550	97.45	97.34	99.12
LiteNet	454	97.87	97.78	99.33

**Table 7:** Comparison of four CNN-based networks for set B with the ten-fold cross-validation method.

Network	PC	ACC (%)	AUC (%)	F1-Measure (%)
AlexNet	1100	98.83	99.05	99.68
GoogleNet	1276	99.01	99.24	99.53
SqueezeNet	528	98.29	98.92	99.41
MobileNets	550	98.20	98.82	99.28
LiteNet	454	98.80	99.30	99.66
